# Docking Applied to the Prediction of the Affinity of Compounds to P-Glycoprotein

**DOI:** 10.1155/2014/358425

**Published:** 2014-05-27

**Authors:** Pablo H. Palestro, Luciana Gavernet, Guillermina L. Estiu, Luis E. Bruno Blanch

**Affiliations:** ^1^Medicinal Chemistry, Department of Biological Sciences, Faculty of Exact Sciences, National University of La Plata, 47 and 115, B1900BJW La Plata, Argentina; ^2^Department of Chemistry and Biochemistry and the Center for Rare and Neglected Diseases, University of Notre Dame, Notre Dame, IN 46556-5670, USA

## Abstract

P-glycoprotein (P-gp) is involved in the transport of xenobiotic compounds and responsible for the decrease of the drug accumulation in multi-drug-resistant cells. In this investigation we compare several docking algorithms in order to find the conditions that are able to discriminate between P-gp binders and nonbinders. We built a comprehensive dataset of binders and nonbinders based on a careful analysis of the experimental data available in the literature, trying to overcome the discrepancy noticeable in the experimental results. We found that Autodock Vina flexible docking is the best choice for the tested options. The results will be useful to filter virtual screening results in the rational design of new drugs that are not expected to be expelled by P-gp.

## 1. Introduction

Human P-glycoprotein (P-gp) belongs to a super family of membrane proteins involved in transport known as ATP binding cassette (ABC) family [1]. It is expressed in normal organs that are important for the absorption-elimination-distribution processes for drugs and xenobiotics [2]. Via P-gp, endogenous and xenobiotic compounds are transported across membranes against the concentration gradient. They flow out of the cell at expenses of the energy associated with the hydrolysis of the cytosolic ATP domains, attached to the transmembrane domains of the protein. This export mechanism allows the P-gp (and other related ABC proteins) to detoxify cells by preventing exogenous compounds from entering susceptible organs [2].

In contrast to this beneficial effect, P-gp may affect negatively the pharmacokinetic profile of new drugs, which has to be discarded in the preclinical stages of drug discovery. P-gp has been implicated in cellular resistance to anticancer drugs, which is believed to be originated in a lowering of the concentration of many anticancer drugs in tumor cells, although the level of the diminution due to the P-gp action itself remains unclear [3–5]. In this context, current medicinal chemists concentrate their efforts not only in the optimization of the compound activity but also in the improvement of ADME/TOX properties.

Our interest in the P-glycoprotein lies on the design of new anticonvulsant drugs that overcome one of the problems of the current medications: they fail to control the symptoms in around one-third of the patients (condition known as drug resistant epilepsy) [6]. One of the accepted causes of the refractory epilepsy is the limited bioavailability of the drugs in the brain thanks to the overexpression or activation of efflux transporters such as P-glycoprotein [7]. It would be favorable, then, to design new anticonvulsant with no interaction with this glycoprotein.

Computational models constitute a fast and low-cost alternative to detect potential P-gp binders among new entities at the early stages of the drug design. During more than 20 years of research, several* in silico* studies have been oriented to elucidate the structural and physicochemical characteristics required for a chemical compound to be P-gp substrate. Early investigations proposed the existence of planar aromatic rings, basic nitrogen atoms, and lipophilic centers as common characteristics shared by inhibitors and substrates of the protein [8, 17]. Additionally, a pharmacophore model proposed the importance of functional groups capable of hydrogen bonding to P-gp [9]. After these first investigations, other ligand-based approaches were developed to identify new possible P-gp substrates, based on different approaches such as pharmacophoric patterns, machine-learning algorithms, and quantitative structure activity relationship (QSAR) studies among others [10–22].

In general, the ligand-based methods perform well in discriminating binders from nonbinders, and they present the advantage of being faster than target-based methods. For those reasons they constitute a valuable tool to select binders/nonbinders in virtual screening campaigns on large databases. On the other hand, target-based methods deal with the computational cost for virtual screening and need the 3D structure of the target, but they are able to predict the interactions responsible for binding for each ligand.

Recently, two research groups obtained the X-ray structures of eukaryotic (mouse) Apo- and ligand-bound P-gp (PDB codes 3G5U, 3G60, 3G61, 4KSB, 4KSD, and 4LSG) [23, 24], improving enormously the knowledge of the protein structure at atomic level.

**Figure 1:**
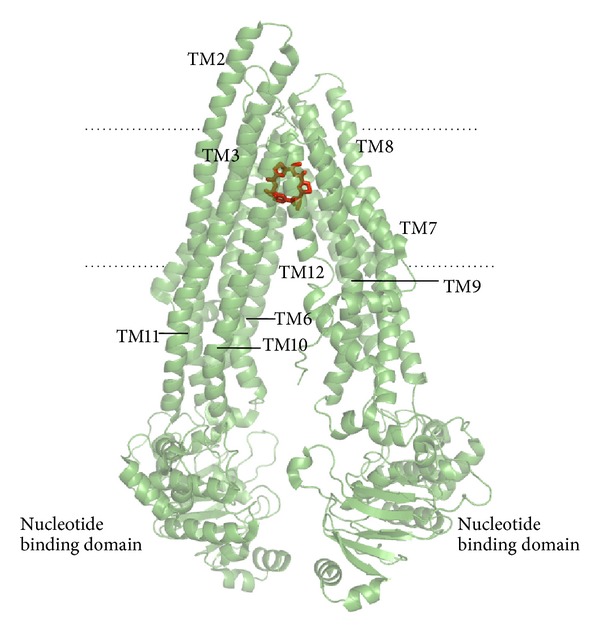
Crystal structure of the complex of mouse P-gp colored in green with its inhibitor in red (pdb code 3G60). Some visible transmembrane domains are labeled (TM). Dashed horizontal lines approximate the region of the lipid bilayer.

As other ABC transporters, P-gp has two transmembrane domains and two cytosolic ATP binding/hydrolysis domains. The experimental structures of mouse P-gp are nucleotide-free and have an inward-facing conformation formed from two sets of six helices (TMs 1–3, 6, 10, 11 and TMs 4, 5, 7–9, 12) (Figure 1) that generates an internal cavity open to both the cytoplasm and the inner leaflet. This cavity has a volume of around 6000 Å^3^, big enough to accommodate more than one drug/substrate simultaneously [23]. This presumptive drug binding pocket comprises mostly hydrophobic and aromatic residues with no clearly defined subsites of binding [23]. When the P-gp structural information became available, several docking based methodologies have attempted to predict the affinity of compounds and metabolites. Chen et al. evaluated the prediction capability of molecular docking by using two drug bound P-gp available structures as receptors [10]. They concluded that the Glide docking software was unable to discriminate clearly substrates from nonsubstrates by using the best score criterion, a fact that was attributed to the polyspecific nature of substrate binding and to the use of only one active binding pocket in the same docking environment [10]. On the other hand, Dolghih and coworkers applied docking protocols to differentiate between a set of 126 binders and a set of 64 nonbinders of P-gp [25]. They also employed the mouse P-gp crystal structure deposited in the Protein Data Bank [23] to model the receptor binding cavity, which was treated as both rigid and flexible. Rigid docking was evaluated using both standard precision Glide and extra precision Glide scores [26]. Flexible receptor docking was implemented using a multistage induced fit docking protocol [27], where the final scoring was performed using the extra precision (XP) Glide scoring function [26] and an MM-GB/SA rescoring function [28–32]. They concluded that the flexible receptor model has the ability to differentiate known binders from nonbinders of P-gp [25]. The authors associated the importance of considering the flexibility of the protein for the identification of P-gp binders and nonbinders by docking with the size and flexibility of the binding cavity.

Bikadi et al. [11] also studied the ability of the models to analyze substrate-P-gp interactions at an atomic level. They stripped and docked the P-gp inhibitors that were cocrystallized in the mouse X-ray Pg-P structures [11]. They found an acceptable agreement between the experimental and predicted ligand conformations, corroborating the capability of the docking calculations to predict P-gp-ligand complex geometry. They constructed a homology model of human P-gp (using the mouse P-gp as template) and successfully docked the Rhodamine B molecule, a P-gp substrate for which the experimental binding data are available. They used Autodock Vina docking software for the simulations and constructed a web server for docking calculations into the structure of mouse P-gp and the homology model of human P-gp, which is free for 10 runs by user [11].

The model from Bikadi et al. has not been used to discriminate binders from nonbinders, as they did not consider docking experiments reliable enough. Nevertheless, this contradicts the results reported by Dolghih using mouse P-gp. Moreover, recent investigations from Dolghih and Jacobson provide an interesting computational approach to differentiate between molecules with high and low efflux ratios as well as CNS-positive and CNS-negative structures based on docking scores. They considered passive permeation and active efflux mediated by P-gp in their approach [33]. Particularly they used the score obtained from docking of mouse P-gp as a measure of the apparent affinity for P-gp, *K*_*m*_, parameter included in the equation to estimate the efflux ratio [33].

Very recently, Klepsch and collaborators used a large set of 1076 P-gp inhibitors and 532 noninhibitors for docking [22]. The ChemScore docking model was able to predict 76% of P-gp inhibitors and 73% of noninhibitors. They also added the log *P* value to the docking score, as a measure of the ability of the compounds to cross the membranes by diffusion. It caused a slight improvement in the prediction of true inhibitors, with values of 0.77 for accuracy and 0.81 for sensitivity (that is, 81% of the inhibitors predicted), at expenses of a decrease in the detection of noninhibitors (0.69 of specificity) [22].

Following the line of these investigations, we present here a study of the ability of the docking software to predict the binding of compounds to human P-gp. As stated before, the final purpose is to have a filter to eliminate compounds that interact with human P-gp for the design of new anticonvulsant compounds to treat refractory epilepsy. To this end, we have built a new dataset of compounds that includes both inhibitors and substrates (binders) and nonbinders carefully selected from the biological results available in the literature. Special attention was given to the classification of the compounds due to the controversy found in the literature (see Section 2).

We used different docking software and conditions and the scores of dockings as the variable to classify the compounds, analyzing the receiver operating characteristic (ROC) [34] curves for each docking model to decide the best docking conditions and choose the limiting score that can be used to discern between substrate and nonsubstrate classes. This information will be useful to decide the conditions for future virtual screening campaigns oriented to identify substrates/inhibitors (and nonsubstrates) of P-gp.

## 2. Methods

### 2.1. P-gp Homology Model

The P-gp sequence was taken from UniProt database (accession code P08183), and the conformation of the human P-gp protein was built using I-TASSER [39, 40] which aligns the query sequence with several templates (known structures) from the protein data bank library. From the homology models generated, the one with highest C-score was selected for further refinement. This alignment was mainly based on 3G61, which provides the highest coverage (0.92). Additionally, we evaluate the ability of I-TASSER to predict the crystal structure of the mouse P-gp (code 3G61) with positive results (see Section 3).

The human homologue was refined by relaxing the predicted structure. The protein atoms were surrounded by a periodic box of TIP3P32 water molecules that extended 10 Å from the P-gp structure with the Tleap module of AMBER11 software [41], and the initial geometry was minimized (1000 cycles for the water molecules followed by 2500 cycles for the entire systems) using the ff03.r1 version of the all-atom AMBER force field [42]. The membrane was not modeled as it will not impact the results of the minimization. We used the Ramachandran plots obtained from the protein structure validation software suite (PSVS) to test the final model [43].

### 2.2. Docking

The dataset of binders and nonbinders was docked in the human P-gp homology model using Glide (version 5.7, Schrodinger Suite 2011), Autodock4.2 [44], and Autodock Vina [45] docking software. In Autodock4.2 the structures were docked using the Lamarckian genetic algorithm in the “docking active site,” defined through a 24 × 24 × 24 Å^3^ grid, centered on the relative position of the ligand in the crystallographic structure (pdb code 3G61). This docking region comprises the entire transmembrane region since the binding subsites reported up to date for substrates and inhibitors reside in the cell membrane, involving residues located in the transmembrane helices [23].

We employed the default grid spacing (0.375 Å) and performed 100 docking runs for each compound, treating the docking active site as a rigid molecule and the ligands as flexible; that is, all nonring torsions were considered active. We also used the default Autodock parameters for all the variables such as Marsilli-Gasteiger partial charges. Additionally, flexible dockings were performed with autodock4.2, using the same parameters and conditions but defining the mobile residues.

Two different sets of amino acids were allowed to move in the flexible docking simulation. In one system we considered the active site residues Phe-335, Phe-343, Phe-728, Phe-732, and Phe-978 as flexible (model A), whereas in the other simulation we considered as flexible Tyr-307, Tyr-953, Phe-343, and Phe-978 (model B). The criterion of selection of the mobile residues in model A was based on the analysis of the amino acids that interact with the experimental ligands in the mouse complexes (codes 3G60 and 3G61). For model B, we examined the conformation of the flexible residues in Model A after the docking simulations. We found that Phe-343 and Phe-978 showed different conformations depending on the ligand, whereas Phe335, Phe732, and Phe728 adopted practically the same conformation in all the tested compounds. Therefore we choose another set of flexible residues that includes Phe-343, Phe-978, and other amino acids that interact with the ligands according to the docking results with model A.

The same protocol was implemented for Autodock Vina. This docking software differs from Autodock in the scoring function, and on average it performs better, both in speed and accuracy [45]. However, we compared both methods as the accuracy is usually dependent on the system (ligand-target), keeping a similar grid size, center, and standard grid spacing as for the case of Autodock. We computed 20 docking runs for each compound using the default parameters for the rest of the variables, comparing also rigid and flexible dockings for the same mobile amino acids defined for the latter. Another docking run was performed with Autodock Vina (for model B) for which we included some additional nonsubstrates in the set (see Section 3).

A standard procedure was used for the Glide docking. The compounds of the set were prepared using the ligprep tool of the Schrodinger 11 suite, in order to generate possible protonation states at different pH and potential low energy conformations. The structure of the protein was processed using the Protein Preparation Wizard tool, performing an exhaustive sampling of the orientations of groups. The protein structure was then refined to relieve steric clashes by means of a restrained minimization with the OPLS2001 force field until a final rmsd of 0.030 Å relative to the input protein coordinates. The prepared structure was used to build the grid, centered as described for Autodock. The single precision (SP) and extra precision (XP) protocols were used in the docking, saving three poses in the SP run to build the library used in the XP run. No constrains were used in the docking, the sampling of ring conformations was included, and nonplanar amide conformations were penalized.

Two different docking runs have been performed, for the compounds in their protonation state at physiological pH and for their neutral form. The latter was modeled taking into account that the binders have been proposed to move from the extra cellular space to the P-gp active site through the membrane, without entering the cell and would be neutral in the lipophilic environment of the membrane [23]. For chiral molecules, all the possible stereoisomers were included.  Table 1 summarizes the different docking conditions.

We also docked the dataset on the X-ray determined mouse structure of P-gpin order to compare and validate the results.

### 2.3. Dataset Preparation: Biological Data Collection

Several biological assays have been developed to test the ability of compounds to interact with P-gp [35, 37]. Among them,* in vitro* transport experiments were recommended by the US Food and Drug Administration as the initial data to decide if a drug is a P-gp substrate/inhibitor. Particularly, they suggested a bidirectional transport assay using cultured cells as the initial test, followed by the validation that the efflux is inhibited by the presence of one or more inhibitors [35]. A recent summary of the* in vitro* methods showed that Caco-2 cells were the most frequently used, followed by the MDCKII-MDR1 cells (multidrug resistance protein 1 transfected–Madin Darby Canine Kidney cells) [36].

The preparation of the datasets with high quality and enough quantity is one of the most important steps for constructing models with high confidence. Special attention was given to the selection and categorization of the compounds, since there is some controversy in the literature regarding the classification of substrates/inhibitors and nonsubstrates/noninhibitors; for example, Polli et al. classify Verapamil as nonsubstrate according to the monolayer efflux experiment in MDCK cells [38], whereas Feng and collaborators consider it as substrate in the same assay [46]. Doan et al. [47] report Fluoxetin as a nonsubstrate (monolayer efflux experiment in MDCKII-MDR1 cells) but in the assay of Calcein-AM (CAM) inhibition (same cellular line) it behaves as an inhibitor [48]. Similar disagreement was found by Feng and collaborators [46]. They found Fluoxetin inactive in the efflux assay and ATPase experiment but showing inhibitory action in the CAM protocol.

In order to prepare a representative and diverse set, the biological results from multiple publications were considered and priority was given to* in vitro* assays over* in vivo* tests.* In vivo* studies deal with complex systems (patients/animals), making it more difficult to evaluate if a new entity is substrate for P-gp or not. Additionally, there is more information available derived from* in vitro* tests, as they better allow the screening of numerous compounds than* in vivo* assays.

After a thorough analysis, we decided to define as “binders” those substrates/inhibitors that were detected in two or more publications in different assays (if possible, more than 3). Conversely, we considered nonbinders those compounds found as nonsubstrates preferably in two or more different assays and not reported as substrate/inhibitors (or reported in only one test). The compounds reported with some controversy in the results (e.g., one test where the compound was considered substrate/inhibitor and another where it behaves as nonsubstrate) were discarded. From this selection 26 compounds were defined as binders and 13 as nonbinders.  Table 2 shows the compounds selected and the classification obtained. The full dataset analyzed is given as supporting information (Table A1 in Supplementary Material available online at http://dx.doi.org/10.1155/2014/358425).

## 3. Results and Discussion

The structure of Human P-gp predicted by I-TASSER and refined by geometry minimization (AMBER) was tested by the protein structure validation software suite (PSVS). Ramachandran plots obtained from the model (Figures A1 and A2 in supporting information) show that the backbone dihedral angles are mainly distributed in allowed regions, and they are similar to the one obtained from the protein data bank for the experimental mouse structure. We also predicted the crystal structure of mouse P-gp (code 3G61) by I-TASSER. The best C-score model fits well with the experimental structure, with a RMSD of 0,65 from the structure alignment (Figure B1). This result increases our confidence of the capacity of I-TASSER to predict the P-GP human structure.

**Figure 2:**
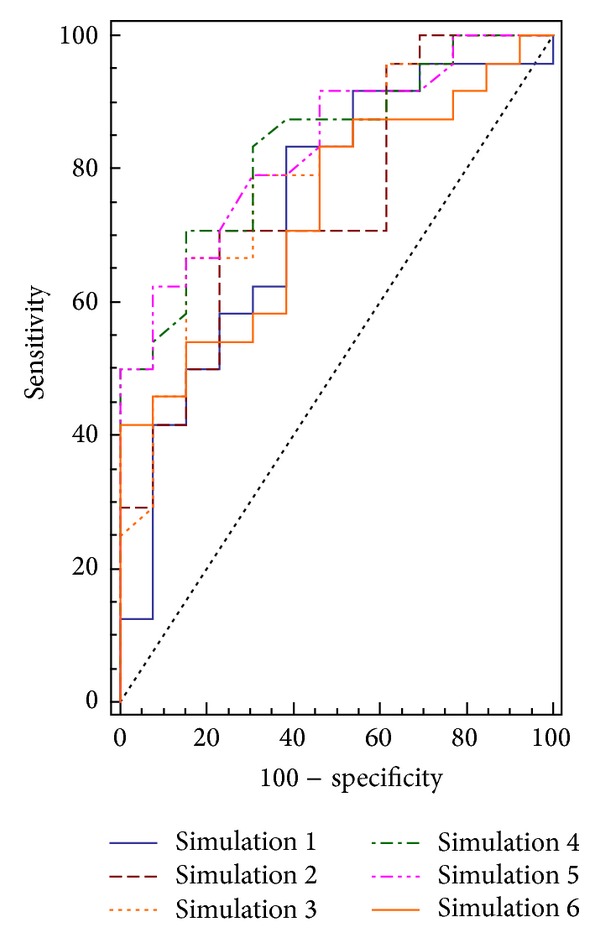
ROC-type curves obtained for the simulations.

With the human protein constructed and the set of compounds defined, we performed the Docking simulations detailed in Table 1.  Figure 2 shows the “receiver operating characteristic” (ROC) curves for each model. The ROC curves graph the true positive rate (sensitivity) as a function of the false positive rate (1−specificity) for all possible threshold levels. Accordingly, the ideal classification would be represented by a line that starts from the origin, reaches vertically the upper left corner, and then goes to the upper right corner. By visual inspection of the curves we found that they are similar, but Autodock Vina scoring function (simulations 4 and 5) looks somewhat better than the others. However, after the comparison of the curves with MedCalc (MedCalc Software, Mariakerke, Belgium) we found that there is not a statistically significant difference between the simulations (95% confidence interval). This prompted us to use the best area under the curve value (AUC) to measure the absolute quality of the simulations (Figure 2).

**Figure 3:**
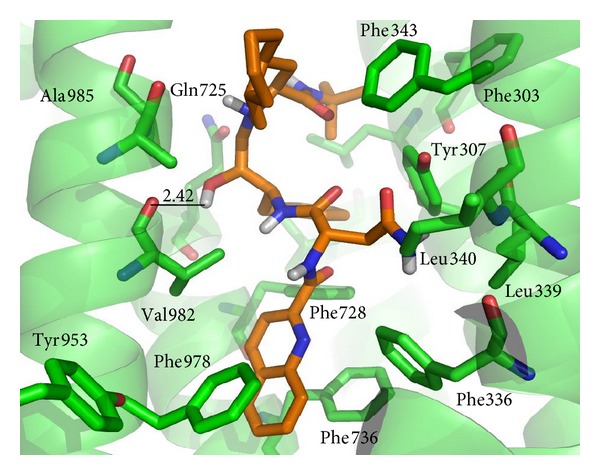
Binding geometry of compound Saquinavir into the P-gp binding pocket predicted by the Autodock docking algorithms (simulation 5). Residues of the binding pocket are highlighted in green. Only the N-bound and O-bound H atoms of the ligand are shown. Carbon atoms of Saquinavir are highlighted in orange. Values of the relevant distances are given in Å.

Autodock Vina simulations 4 and 5 were able to better discriminate between binders and nonbinders. As expected, the best results were obtained using flexible docking (with Tyr-307, Tyr-953, Phe-343, and Phe-978 residues defined as mobile). It becomes interesting that these two docking protocols differ only in the protonation state of the ligands. The observation that the ionization states of the binders are not important for the docking process is consistent with the lipophilic nature of the active site. According to the experimental data, the P-gp drug binding pocket comprises mostly hydrophobic and aromatic residues [23].  Figure 3 shows the docking solution for the binding of Saquinavir to the P-gp active site (one of best scores of the set in simulation 5), as an example of the characteristic interactions found for binders. Saquinavir shows a weak H-bond interaction with Val982 peptide bond, but its binding is mainly stabilized through lipophilic and stacking interactions with the aliphatic and aromatic residues that surround the molecule.

Our findings support the fact that the ionization states of the molecules of the set do not modify substantially their interactions with the binding site (and the capacity to discriminate binders from nonbinders). It may affect the ability of the binders to reach the active site, but this phenomenon cannot be measured during the docking process.

We also docked the dataset into the mouse P-gp structure using Autodock Vina (condition of simulation 5). The AUC obtained (0.821) was similar but lower than the one of the human model. These results provide an additional test to validate our human model.

**Figure 4:**
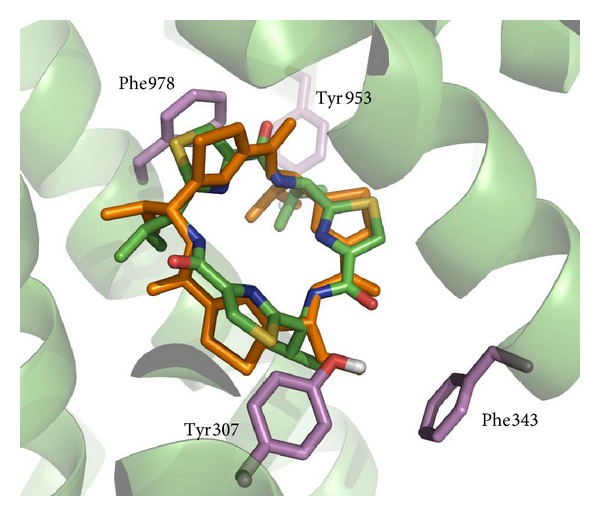
Superimposition of the docking of compound QZ59 (conditions of simulation 5) in the homology model and the mouse X-ray structure of the complex 3G60. Atom carbons of the flexible residues are highlighted in violet. The X-ray structure of QZ59 in 3G60 is shown in orange for comparison.

Additionally, the inhibitor cocrystallized in the mouse experimental structure (ligand named QZ59, pdb code 3G60) was redocked into the homology model (simulation 5) to evaluate the capacity of the model to reproduce the mouse experimental conformation.  Figure 4 shows the accuracy of the model through superimposition of the best conformation obtained from the docking and the experimental structure. On the other hand, docking with the same P-gp model and in the same conditions was performed with the structures from the subset of Zinc database that includes commercially available approved drugs (Zdd) [49]. The best score was found for the drug Telmisartan, a member of a family of drugs called angiotensin receptor blockers [50]. The literature confirmed the positive interaction of this compound with P-gp by means of* in vitro* permeation study in MDR1-MDCK II cell monolayers [50].

The docking simulations mentioned before reveal the capacity of the model to predict P-gp substrates as well to reproduce experimental interactions. However, these results (Figure 2) were obtained using a dataset where the number of nonbinders molecules is less than the number of binders (13 versus 26). As a test of accuracy, we repeated the docking in the conditions of simulation 5 but included in the set 13 endogenous molecules from the KEGG database [51], in order to have the same number of molecules in both subsets used for a ROC curve analysis [34]. The new structures were considered as nonbinders based on the concept that their expulsion from the cell would be inefficient to its function. This database was used with the same criterion by other authors to increase the number of nonbinders [24]. As we need only 13 structures to equilibrate the dataset, we selected from the database the structures with similar molecular weight compared with the values of the other compounds of the dataset. Small molecules tend to be scored worse by scoring functions, causing a favorable error in the value of AUC.

**Figure 5:**
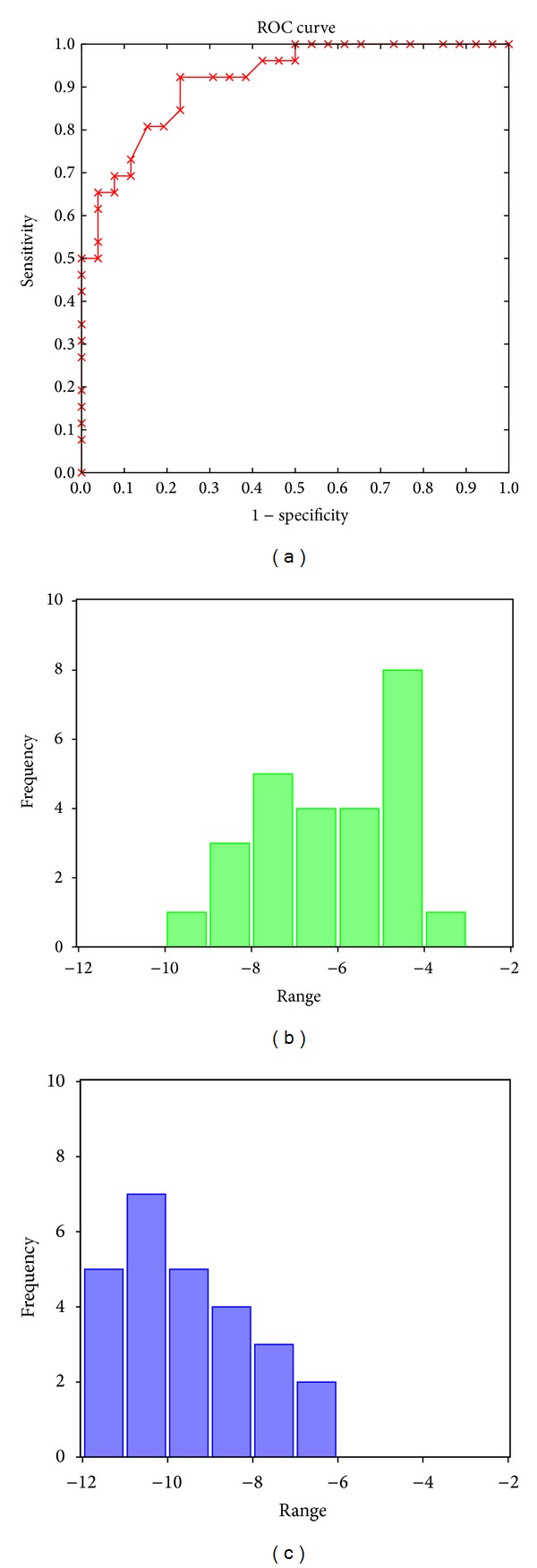
ROC-type curve and score distribution using the conditions of simulation 5 for the compounds of the extended set that includes metabolites. Binders are represented in blue whereas nonbinders in green.

The ROC curve of the resulting simulation is shown in Figure 5, with an AUC of 0.916 (best threshold of −7.4), which is better than the previous one reported in Table 1. Figure 5 also shows the distribution of scores for binders and nonbinders. This model is able to predict the 85% of the binders (sensitivity value of 0.85) and the 77% of nonbinders (specificity value of 0.77) with a global accuracy value of 0.81. Although it is not expected to find a linear relationship between the value of the scores and the experimental data of P-gp efflux, we found that the last model assigns a more favorable value (more negative) to binders than to nonbinders (Figure 5).

**Figure 6:**
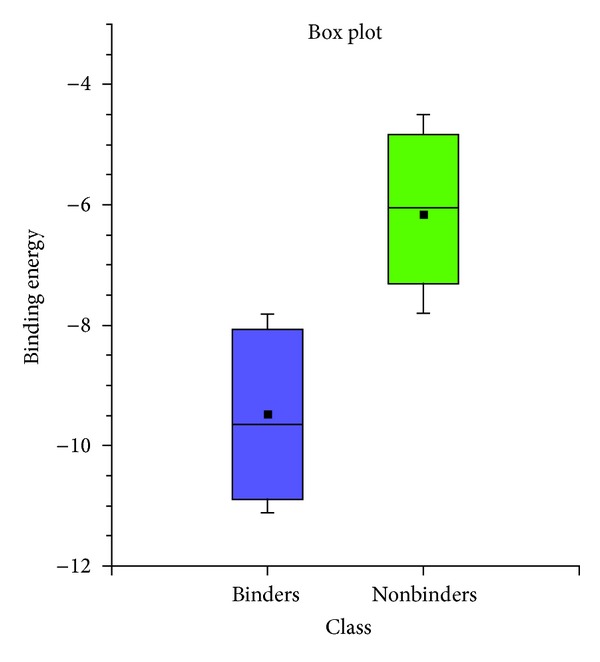
Box plots that show the Q3–Q1 interquartile range for P-gp binders (in blue) and nonbinders (in green). The ends of the whiskers represent the standard deviation above and below the mean of the data. Median value is represented by a line and mean value as a square.

For future virtual screening campaigns, we adopted the median value of each set to define a threshold value for binders and nonbinders. The median is defined as the central datum of each subset when the data are arranged in numerical order.  Figure 6 shows the box and whiskerplots obtained for binders and nonbinders, showing a clear separation between both subsets. The boxes represent the interquartile range Q3–Q1 and the band inside the box is the median value. The ends of the whiskers represent the standard deviation above and below the mean of the data, which is shown as a square inside the box. Therefore, a docking score of −9.7 or lower would be indicative of an effective interaction with Pg-p. Conversely, a score of −6.1 or higher would predict nonaffinity to the P-gp active site.

In relation to the performance of the model, it presents a better predictive capacity than other docking protocols recently reported by Klepsch and coworkers (see Section 1), but they are less efficient than ligand-based methods proposed by the same authors (with values around 0.9 for sensitivity and 0.86 for the overall accuracy for the best model) [22]. Since the docking force fields involve “pure” ligand-based terms to calculate the binding energy for each ligand, we analyzed the contribution of the ligand internal energy to the final score for Autodock simulations. We found that the internal energy of the ligands varies from 0 to 36.5% of the final score (Table B1). As expected, the contribution of the ligand-based terms is more important when the structures have rotatable bonds. We did not find a clear correlation between the internal energy of the ligands and the final classification of binders and nonbinders, showing the importance of ligand-receptor interaction based terms for the prediction of P-gp binding. We believe that the docking protocol presented here could be useful for designing new anticonvulsant drugs (or other active compounds) with no interaction with P-gp. It is able to discriminate reasonably well between binders and nonbinders and provide additional information related to the ligand-Pgp interactions responsible for binding. This last information would be useful for designing new related structures that preserve the therapeutic action but avoid the interactions with the glycoprotein.

## 4. Summary

The* in silico* prediction of positive interactions of structures with the human P-gp active site constitutes a valuable tool for the design of new P-gp inhibitors. In the same line, the early recognition of structures with low interaction with the glycoprotein and high affinity for other specific targets is important for the design of new drugs and/or the discovery of new leaders. In this investigation we present a flexible docking protocol with the capacity to distinguish binders from nonbinders from a representative set of compounds.

Our future investigations are directed to apply this procedure as a filter at the beginning of the virtual screening path when searching for new anticonvulsant drugs. As epilepsy is a disorder with high prevalence of drug resistance, the early recognition of substrates of P-gp will allow us to avoid the selection of candidates with poor bioavailability in the brain. Some of the results obtained from the use of this* in silico* P-gp filter are already published [52] since they were acquired during the writing of this paper.

## Supplementary Material

The Supplementary Material includes Ramachandran plots of the human model of the P-glycoprotein. It also includes a figure of the superimposition of the predicted and the crystal structure of mouse P-gp, a table with the full dataset of binders and non-binders analyzed and the contribution of the ligand internal energy to the final docking score.

## Figures and Tables

**Table 1:** Conditions and characteristics of the docking simulations performed.

Software	Flexible residues	Protonation state of the ligands	Name of the simulation	AUC
Autodock4.2	Model A	Neutral	Simulation 1	0.737
Autodock4.2	Model B	Neutral	Simulation 2	0.747
Autodock Vina	Model A	Neutral	Simulation 3	0.790
Autodock Vina	Model B	Neutral	Simulation 4	0.833
Autodock Vina	Model B	pH 7.4	Simulation 5	0.833
Glide	Rigid	All possible	Simulation 6	0.737

**Table 2:** Structures that comprise the dataset. The references of the biological data are indicated in square bracket for each compound. N means that the selected compound is negative against the biological assay whereas P means that it results positive in the test.

Compound	Biological assays				
	Monolayer efflux	ATPase	Calcein-AM	Rhodamine-123	*In vivo*
Amantadine	N [35, 36]	N [35]	N [35, 36]		
Carbamazepine	N [6, 36, 37]	N [37]	N [36, 37]	N [6]	N [6]
Chlorpheniramine	N [35, 36]	N [35]	N [35, 36]		
Ethosuximide	N [6, 37]	N [37]	N [37]		
Felbamate				N [6]	
Fluvoxamine	N [36, 37]	N [37]	N [37] P [36]		
Lidocaine	N [35, 36]	N [35]	N [35, 36]		
Mannitol	N [35, 36]	N [35]	N [35, 36]		
Propanolol	N [35, 36]	N [35]	N [35, 36]		
Ranitidine	N [35]	N [35, 38]	N [35, 38]	N [38]	
Sumatriptan	N [35, 36]	N [35]	N [35, 36]		
Trazodone	N [36, 37]	N [37]	N [37]P [36]		
Vigabatrin	N [6]				N [6]
Amprenavir	P [35, 36]	P [35]	P [35, 36]		
Astemizole	P [36]	P [38]	P [36, 38]	P [38]	
Cyclosporine	P [35]	N [35] P [38]	P [35, 38]	P [38]	
Dexamethasone	P [35, 38]	N [35] P [38]	N [35, 38]	N [38]	
Diltiazem	P [35, 36]	P [35]	P [35, 36]		
Indinavir	P [35, 36]	P [35]	N [35, 36]		
Ketoconazole	N [35]	P [35, 36]	P [35, 36]	P [32]	
Lamotrigine	N [6, 37] P [6]	N [37]	N [37]	P [6]	P [6]
Levetiracetam	N [6]P [6]			P [6]	P [6]
Loperamide	P [35–37]	P [35, 37]	P [35–37]		
Loratadine	P [35] N [37]	P [35, 37]	P [35, 37]		
Nelfinavir	P [35]	P [35, 38]	P [35, 38]	P [38]	
Neostigmine	P [35, 36]	N [35]	N [35, 36]		
Nicardipine	N [35]	P [35, 38]	P [35, 38]	P [38]	
Oxcarbazepine	P [6]				P [6] N [6]
Phenobarbital	P [6] N [6]	P [6]		P [6]	P [6]
Phenytoin	P [6, 37] N [6]	N [37]	N [37]	N [6]	P [6]
Prazosin	P [35, 37]	P [35, 37]	N [35, 37]		
Quinidine	P [35, 37]	P [35, 37]	P [35, 37]		
Risperidone	P [36, 37]	P [37]	P [36] N [37]		
Ritonavir	P [35, 37]	P [35, 37, 38]	P [35, 38] N [37, 38]	P [38]	
Saquinavir	P [35, 38]	P [35, 38]	P [35, 38] N [38]	P [38]	
Terfenadine	P [35]	P [35] N [38]	P [35, 38]	N [38]	
Trimethoprim	P [35, 36]	P [35]	N [35, 36]		
Verapamil	P [37, 38] N [35, 37]	P [35, 37, 38]	P [35, 37, 38]	P [38]	
Vinblastine	P [35]	P [35, 38]	P [35, 38] N [38]	P [38]	

## References

[1] Thiebaut F, Tsuruo T, Hamada H, Gottesman MM, Pastan I, Willingham MC (1987). Cellular localization of the multidrug-resistance gene product P-glycoprotein in normal human tissues. *Proceedings of the National Academy of Sciences of the United States of America*.

[2] Szakács G, Váradi A, Özvegy-Laczka C, Sarkadi B (2008). The role of ABC transporters in drug absorption, distribution, metabolism, excretion and toxicity (ADME-Tox). *Drug Discovery Today*.

[3] Juliano RL, Ling V (1976). A surface glycoprotein modulating drug permeability in Chinese hamster ovary cell mutants. *Biochimica et Biophysica Acta*.

[4] Gottesman MM, Pastan I (1988). The multidrug transporter, a double-edged sword. *The Journal of Biological Chemistry*.

[5] Hennessy M, Spiers JP (2007). A primer on the mechanics of P-glycoprotein the multidrug transporter. *Pharmacological Research*.

[6] Zhang C, Kwan P, Zuo Z, Baum L (2012). The transport of antiepileptic drugs by P-glycoprotein. *Advanced Drug Delivery Reviews*.

[7] Talevi A, Bruno-Blanch LE (2013). Pharmacoresistance in epilepsy. *From Genes and Molecules to Promising Therapies*.

[8] Zamora JM, Pearce HL, Beck WT (1988). Physical-chemical properties shared by compounds that modulate multidrug resistance in human leukemic cells. *Molecular Pharmacology*.

[9] Seelig A (1998). A general pattern for substrate recognition by P-glycoprotein. *European Journal of Biochemistry*.

[10] Chen L, Li Y, Yu H, Zhang L, Hou T (2012). Computational models for predicting substrates or inhibitors of P-glycoprotein. *Drug Discovery Today*.

[11] Bikadi Z, Hazai I, Malik D (2011). Predicting P-glycoprotein-mediated drug transport based on support vector machine and three-dimensional crystal structure of P-glycoprotein. *PLoS ONE*.

[12] di Ianni M, Talevi A, Castro EA, Bruno-Blanch LE (2011). Development of a highly specific ensemble of topological models for early identification of P-glycoprotein substrates. *Journal of Chemometrics*.

[13] Ecker GF, Stockner T, Chiba P (2008). Computational models for prediction of interactions with ABC-transporters. *Drug Discovery Today*.

[14] Demel MA, Kraemer O, Ettmayer P, Haaksma E, Ecker GF (2010). Ensemble rule-based classification of substrates of the human ABC-transporter ABCB1 using simple physicochemical descriptors. *Molecular Informatics*.

[15] Cianchetta G, Singleton RW, Zhang M (2005). A pharmacophore hypothesis for P-glycoprotein substrate recognition using GRIND-based 3D-QSAR. *Journal of Medicinal Chemistry*.

[16] Langer T, Eder M, Hoffmann RD, Chiba P, Ecker GF (2004). Lead identification for modulators of multidrug resistance based on in silico screening with a pharmacophoric feature model. *Archiv der Pharmazie*.

[17] Pearce HL, Safa AR, Bach NJ, Winter MA, Cirtain MC, Beck WT (1989). Essential features of the P-glycoprotein pharmacophore as defined by a series of reserpine analogs that modulate multidrug resistance. *Proceedings of the National Academy of Sciences of the United States of America*.

[18] Sakiyama Y (2009). The use of machine learning and nonlinear statistical tools for ADME prediction. *Expert Opinion on Drug Metabolism & Toxicology*.

[19] Wang Y-H, Li Y, Yang S-L, Yang L (2005). Classification of substrates and inhibitors of P-glycoprotein using unsupervised machine learning approach. *Journal of Chemical Information and Modeling*.

[20] Broccatelli F, Carosati E, Neri A (2011). A novel approach for predicting P-glycoprotein (ABCB1) inhibition using molecular interaction fields. *Journal of Medicinal Chemistry*.

[21] Chen L, Li Y, Zhao Q, Peng H, Hou T (2011). ADME evaluation in drug discovery. 10. Predictions of P-glycoprotein inhibitors using recursive partitioning and naive bayesian classification techniques. *Molecular Pharmaceutics*.

[22] Klepsch F, Poongavanam V, Ecker GF (2014). Ligand and structure-based classification models for Prediction of P-glycoprotein inhibitors. *Journal of Chemical Information and Modeling*.

[23] Aller SG, Yu J, Ward A (2009). Structure of P-glycoprotein reveals a molecular basis for poly-specific drug binding. *Science*.

[24] Ward AB, Szewczyk P, Grimard V (2013). Structures of P-glycoprotein reveal its conformational flexibility and an epitope on the nucleotide-binding domain. *Proceedings of the National Academy of Sciences of the United States of America*.

[25] Dolghih E, Bryant C, Renslo AR, Jacobson MP (2011). Predicting binding to P-glycoprotein by flexible receptor docking. *PLoS Computational Biology*.

[26] Friesner RA, Murphy RB, Repasky MP (2006). Extra precision glide: docking and scoring incorporating a model of hydrophobic enclosure for protein-ligand complexes. *Journal of Medicinal Chemistry*.

[27] Sherman W, Day T, Jacobson MP, Friesner RA, Farid R (2006). Novel procedure for modeling ligand/receptor induced fit effects. *Journal of Medicinal Chemistry*.

[28] Ghosh A, Rapp CS, Friesner RA (1998). Generalized born model based on a surface integral formulation. *Journal of Physical Chemistry B*.

[29] Huang N, Kalyanaraman C, Irwin JJ, Jacobson MP (2006). Physics-based scoring of protein—ligand complexes: enrichment of known inhibitors in large-scale virtual screening. *Journal of Chemical Information and Modeling*.

[30] Kollman PA, Massova I, Reyes C (2000). Calculating structures and free energies of complex molecules: combining molecular mechanics and continuum models. *Accounts of Chemical Research*.

[31] Wang JM, Hou T, Xu X (2006). Recent advances in free energy calculations with a combination of molecular mechanics and continuum models. *Current Computer-Aided Drug Design*.

[32] Hou T, Li N, Li Y, Wang W (2012). Characterization of domain-peptide interaction interface: prediction of SH3 domain-mediated protein-protein interaction network in yeast by generic structure-based models. *Journal of Proteome Research*.

[33] Dolghih E, Jacobson MP (2013). Predicting efflux ratios and blood-brain barrier penetration from chemical structure: combining passive permeability with active efflux by P-glycoprotein. *ACS Chemical Neuroscience*.

[34] Rizzi A, Fioni A (2008). Virtual screening using PLS discriminant analysis and ROC curve approach: an application study on PDE4 inhibitors. *Journal of Chemical Information and Modeling*.

[35] Ambudkar SV, Dey S, Hrycyna CA, Ramachandra M, Pastan I, Gottesman MM (1999). Biochemical, cellular, and pharmacological aspects of the multidrug transporter. *Annual Review of Pharmacology and Toxicology*.

[36] Agarwal S, Arya V, Zhang L (2013). Review of P-gp inhibition data in recently approved new drug applications: utility of the proposed [I1]/IC50 and [I2]/IC50 criteria in the p-gp decision tree. *Journal of Clinical Pharmacology*.

[37] Giacomini KM, Huang S-M, Tweedie DJ (2010). Membrane transporters in drug development. *Nature Reviews Drug Discovery*.

[38] Polli JW, Wring SA, Humphreys JE (2001). Rational use of in vitro P-glycoprotein assays in drug discovery. *Journal of Pharmacology and Experimental Therapeutics*.

[39] Zhang Y (2008). I-TASSER server for protein 3D structure prediction. *BMC Bioinformatics*.

[40] Roy A, Kucukural A, Zhang Y (2010). I-TASSER: a unified platform for automated protein structure and function prediction. *Nature Protocols*.

[41] Case DA, Darden TA, Cheatham TE (2010). *Amber 11*.

[42] Yang L, Tan C-H, Hsieh M-J (2006). New-generation Amber united-atom force field. *Journal of Physical Chemistry B*.

[43] Bhattacharya A, Tejero R, Montelione GT (2007). Evaluating protein structures determined by structural genomics consortia. *Proteins: Structure, Function and Genetics*.

[44] Morris GM, Goodsell DS, Halliday RS (1998). Automated docking using a Lamarckian genetic algorithm and an empirical binding free energy function. *Journal of Computational Chemistry*.

[45] Trott O, Olson AJ (2010). AutoDock Vina: improving the speed and accuracy of docking with a new scoring function, efficient optimization, and multithreading. *Journal of Computational Chemistry*.

[46] Feng B, Mills JB, Davidson RE (2008). In vitro P-glycoprotein assays to predict the in vivo interactions of P-glycoprotein with drugs in the central nervous system. *Drug Metabolism and Disposition*.

[47] Doan KMM, Humphreys JE, Webster LO (2002). Passive permeability and P-glycoprotein-mediated efflux differentiate central nervous system (CNS) and non-CNS marketed drugs. *Journal of Pharmacology and Experimental Therapeutics*.

[48] Schwab D, Fischer H, Tabatabaei A, Poli S, Huwyler J (2003). Comparison of in vitro P-glycoprotein screening assays: recommendations for their use in drug discovery. *Journal of Medicinal Chemistry*.

[49] Irwin JJ, Sterling T, Mysinger MM, Bolstad ES, Coleman RG (2012). ZINC: a free tool to discover chemistry for biology. *Journal of Chemical Information and Modeling*.

[50] Kataoka M, akashima T, Shingaki T (2012). Dynamic analysis of GI absorption and hepatic distribution processes of telmisartan in rats using positron emission tomography. *Pharmaceutical Research*.

[51] Kanehisa M, Goto S, Furumichi M, Tanabe M, Hirakawa M (2009). KEGG for representation and analysis of molecular networks involving diseases and drugs. *Nucleic Acids Research*.

[52] di Ianni ME, Enrique AV, Palestro PH, Gavernet L, Talevi A, Bruno-Blanch LE (2012). Several new diverse anticonvulsant agents discovered in a virtual screening campaign aimed at novel antiepileptic drugs to treat refractory epilepsy. *Journal of Chemical Information and Modeling*.

