# Supplementary material for: Docking Applied to the Prediction of the Affinity of Compounds to P-Glycoprotein
Source: Biomed Res Int. 2014 May 27;2014:358425. doi: 10.1155/2014/358425 (PMC4058261; doi:10.1155/2014/358425)
Supplement: Supplementary file 1 — The Supplementary Material includes Ramachandran plots of the human model of the P-glycoprotein. It also includes a figure of the superimposition of the predicted and the crystal structure of mouse P-gp, a table with the full dataset of binders and non-binders analyzed and the contribution of the ligand internal energy to the final docking score. [file 358425.f1.zip › Supporting Information/FigureA1.pdf]

PROCHECK-NMR

Ramachandran Plot
pgp_human-itasser_noHs (1 models)

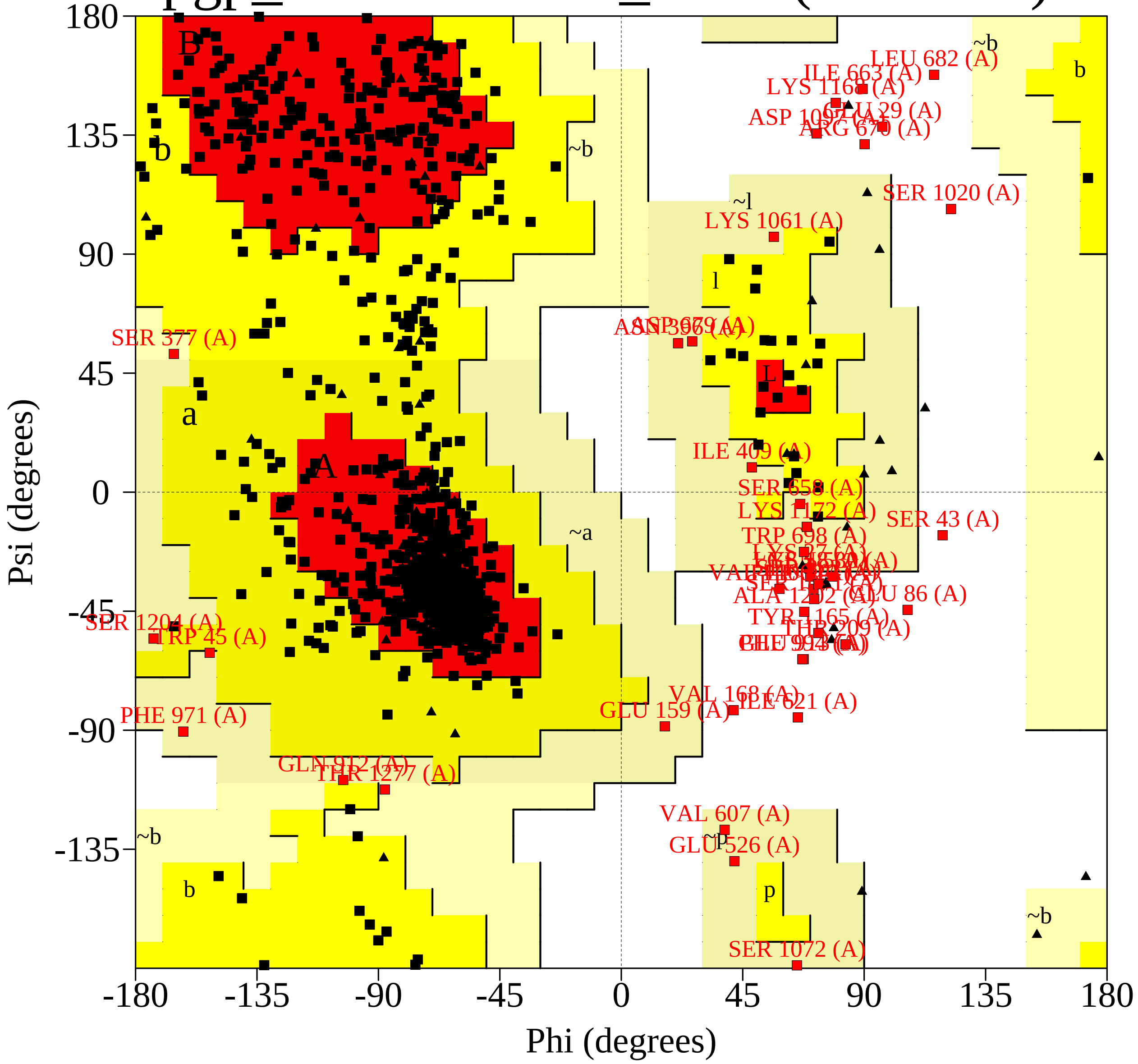

Plot statistics

Residues in most favoured regions [A,B,L]	895	81.5%
Residues in additional allowed regions [a,b,l,p]	163	14.8%
Residues in generously allowed regions [~a,~b,~l,~p]	18	1.6%
Residues in disallowed regions	22	2.0%
	----	------
Number of non-glycine and non-proline residues	1098	100.0%
Number of end-residues (excl. Gly and Pro)	35	
Number of glycine residues (shown as triangles)	99	
Number of proline residues	29	

Total number of residues	1261	

Based on an analysis of 118 structures of resolution of at least 2.0 Angstroms and R-factor no greater than 20%, a good quality model would be expected to have over 90% in the most favoured regions.
