# Supplementary figures and images for: Docking Applied to the Prediction of the Affinity of Compounds to P-Glycoprotein
Source: Biomed Res Int. 2014 May 27;2014:358425. doi: 10.1155/2014/358425 (PMC4058261; doi:10.1155/2014/358425)

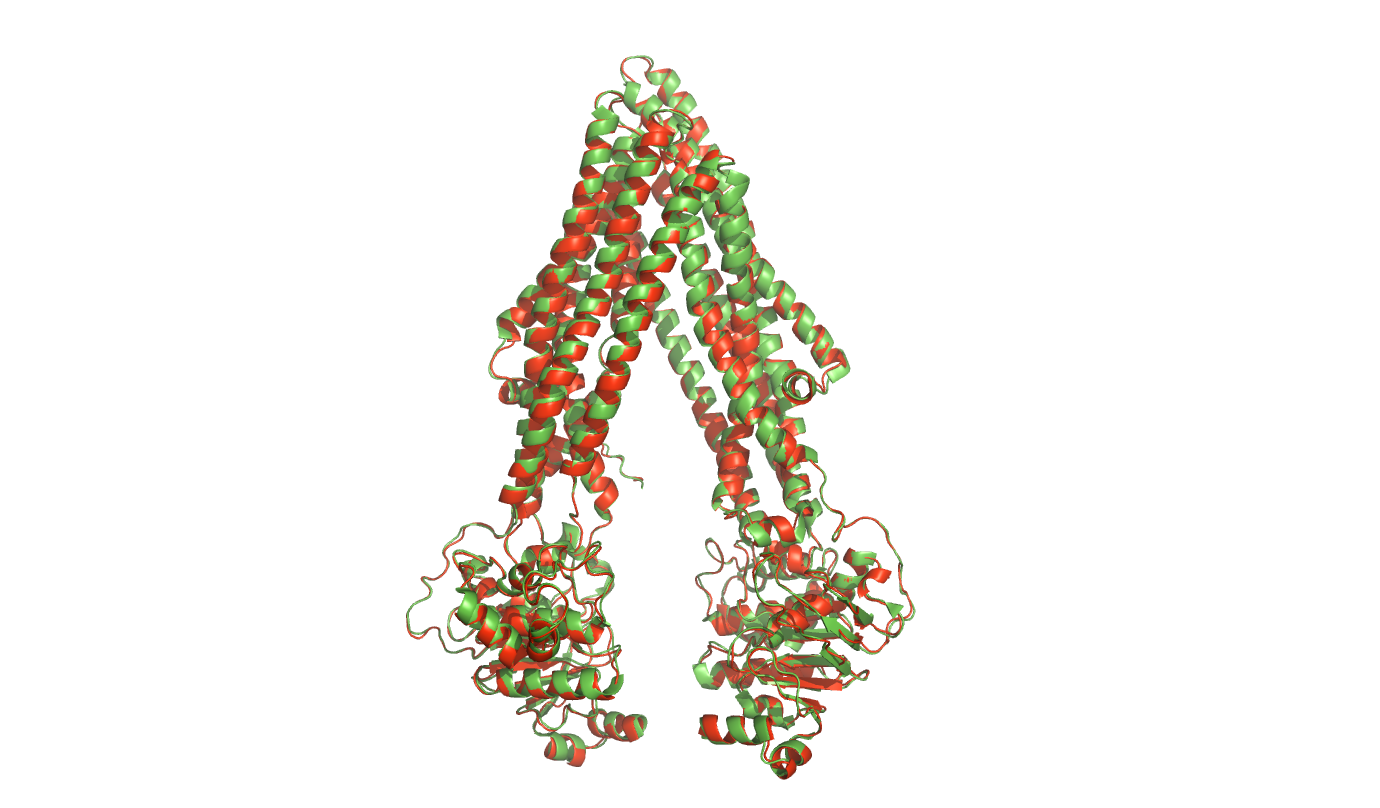

Supplement: Supplementary file 1 — The Supplementary Material includes Ramachandran plots of the human model of the P-glycoprotein. It also includes a figure of the superimposition of the predicted and the crystal structure of mouse P-gp, a table with the full dataset of binders and non-binders analyzed and the contribution of the ligand internal energy to the final docking score. [file 358425.f1.zip › Supporting Information/Figure B1.png]

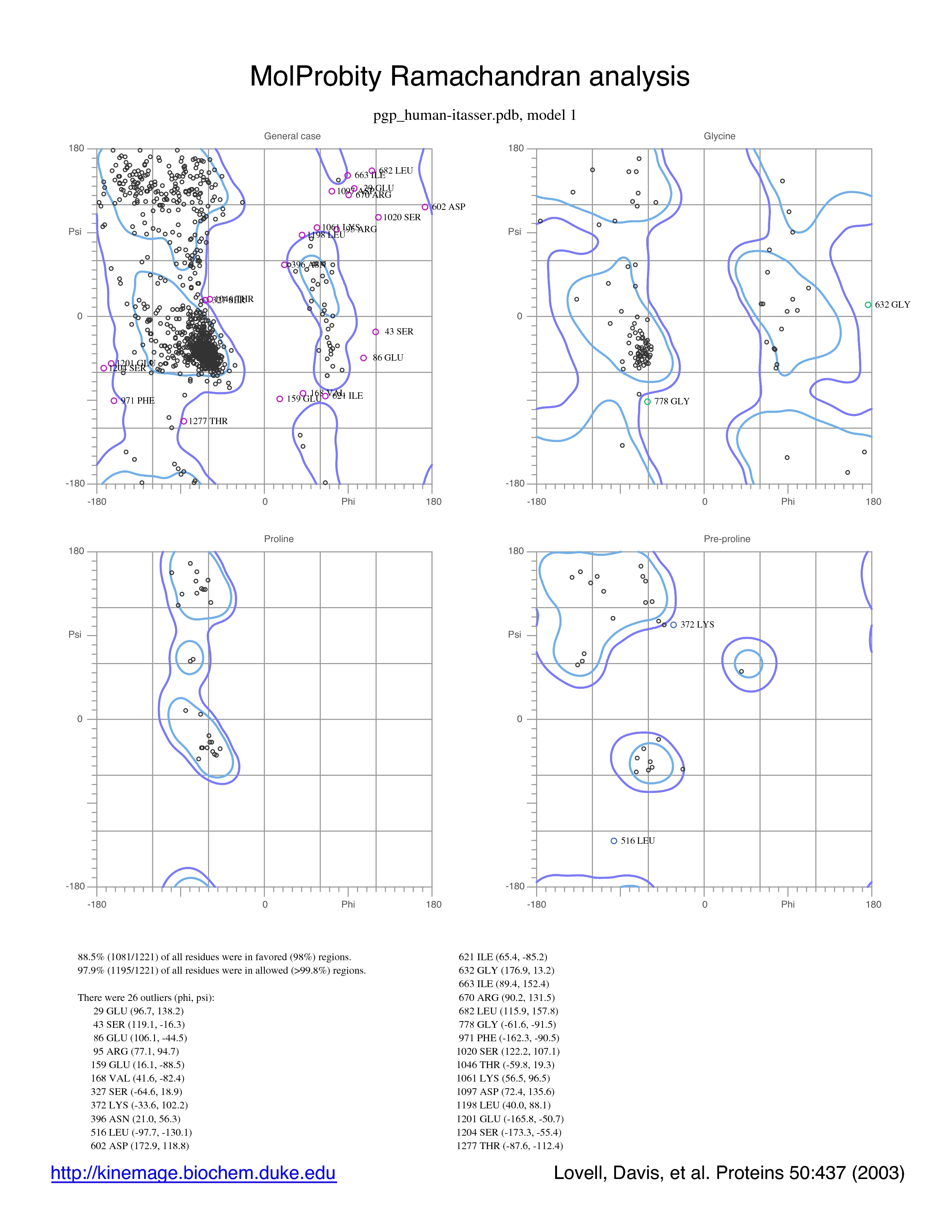

Supplement: Supplementary file 1 — The Supplementary Material includes Ramachandran plots of the human model of the P-glycoprotein. It also includes a figure of the superimposition of the predicted and the crystal structure of mouse P-gp, a table with the full dataset of binders and non-binders analyzed and the contribution of the ligand internal energy to the final docking score. [file 358425.f1.zip › Supporting Information/FigureA2.jpg]
